# Targeting Strategies for Enhancing Paclitaxel Specificity in Chemotherapy

**DOI:** 10.3389/fcell.2021.626910

**Published:** 2021-03-29

**Authors:** Yuan Ma, Sifan Yu, Shuaijian Ni, Baoxian Zhang, Angela Chun Fai Kung, Jin Gao, Aiping Lu, Ge Zhang

**Affiliations:** ^1^Law Sau Fai Institute for Advancing Translational Medicine in Bone and Joint Diseases, School of Chinese Medicine, Hong Kong Baptist University, Kowloon, Hong Kong; ^2^Institute of Integrated Bioinfomedicine and Translational Science, School of Chinese Medicine, Hong Kong Baptist University, Kowloon, Hong Kong; ^3^Institute of Precision Medicine and Innovative Drug Discovery, HKBU Institute for Research and Continuing Education, Shenzhen, China; ^4^Increasepharm and Hong Kong Baptist University Joint Centre for Nucleic Acid Drug Discovery, Hong Kong Science Park, New Territories, Hong Kong; ^5^Increasepharm (Hong Kong) Limited, Hong Kong Science Park, Shatin, Hong Kong; ^6^Increasepharm (Hengqin) Institute Co. Limited, Zhuhai, China

**Keywords:** paclitaxel, ligand, small molecule, antibody, aptamer

## Abstract

Paclitaxel (PTX) has been used for cancer treatment for decades and has become one of the most successful chemotherapeutics in the clinic and financially. However, serious problems with its use still exist, owing to its poor solubility and non-selective toxicity. With respect to these issues, recent advances have addressed the water solubility and tumor specificity related to PTX application. Many measures have been proposed to remedy these limitations by enhancing tumor recognition via ligand-receptor-mediated targeting as well as other associated strategies. In this review, we investigated various kinds of ligands that have emerged as PTX tumor-targeting tools. In particular, this article highlights small molecule-, protein-, and aptamer-functionalized conjugates and nanoparticles (NPs), providing a promising approach for PTX-based individualized treatment prospects.

## Introduction

Paclitaxel (PTX) is a natural terpenoid bearing a tricyclic skeleton. It is an alkaline power taxine after isolation from the leaves of the *European yew*, formerly performed by German scientist Lucas in 1865. PTX was registered as the trademark Taxol^TM^ in 1992 by Bristol–Myers Squibb Company (Weaver, 2014) and approved by the US Food and Drug Administration (FDA) for the treatment of gastric cancer. Due to the increasing demands of the market, breakthroughs in synthetic methodologies have significantly accelerated PTX development. To date, several PTX agents have entered into different stages of clinical trials (shown in Table 1) and have been widely used for the treatment of gastric cancer, breast cancer, ovarian cancer, melanoma, lung cancer, head and neck cancer, and colorectal cancer. In addition, PTX has shown gratifying therapeutic efficacy in heart disease, skin disease, kidney and liver fibrosis, inflammation, and acquired immunodeficiency syndrome (AIDS) (Ibrahim et al., 2002; Kinoshita et al., 2014).

**TABLE 1:** PTX anticancer agents in clinical trials.

**Drug name**	**Formulation**	**Excipients**	**Benefit**	**Status**	**References**
Taxol	Solution	CrEL, absolute ethanol	Effective	Approved internationally	Cordell et al., 1991
Abraxane	Albumin nanoparticle	HSA	Good aqueous solubility, wide scope of application	Approved internationally	Park et al., 2018
PICN	Polymeric nanoparticle	PVP, cholesteryl sulfate, caprylic acid	Decreased corticosteroid pretreatment	Approved in India	Jain et al., 2016
DHP-107	Emulsion	Monoolein, tricaprylin, tween 80	Oral administration	Approved in South Korea	Jang et al., 2017
Lipusu	Liposome	EPC, chol	No immunogenicity	Approved in China	Ye et al., 2013
Cynviloq	Micelle	mPEG-PDLA	Good tolerance dose, high biocompatibility	Approved in South Korea	Le et al., 2018
Paclical	Micelle	XR-17	High drug loading (1.3: 1)	Approved in Russia, Kazakhstan	Bernabeu et al., 2017
NK105	Micelle	PEG-PASA	Better PSN toxicity profile than PTX.	Phase III	Fujiwara et al., 2019
ANG1005	Conjugate	ANG modification	BBB-penetrating	Phase II/III	Régina et al., 2008
Taxoprexin	Conjugate	DHA modification	Modest activity	Phase II/III	Bedikian et al., 2011
Xyotax	Conjugate	PGA modification	Good aqueous solubility, less toxicity	Phase II/III	Singer, 2005
LEP-ETU	Liposome	EPC, cardiolipin, Chol, α-TAS	Better tolerated than taxol	Phase II	Tan et al., 2006
EndoTAG-1	Liposome	DOPC, DOTAP	Effective and good tolerance dose	Phase II	Christopeit et al., 2008
FID-007	Micelle	PEOx	Less toxicity and better efficacy	Phase I	Yin et al., 2013

*CrEL represents Cremophor EL; HAS represents human serum albumin; PVP represents polyvinylpyrrolidone; EPC represents egg lecithin; Chol represents cholesterol; mPEG represents methoxy poly(ethylene glycol); PDLA represents poly(D-lactide); XR-17 consists of two derivatives of vitamin A; PEG represents poly(ethylene glycol); PASA represents polyaspartic acid; ANG represents Angiopep-2; DHA represents docosahexaenoic acid; PGA represents polyglutamic acid; α-TAS represents alpha-tocopheryl acid succinate; DOPC represents 1,2-dioleoyl-sn-glycero-3-phosphocholine; DOTAP represents 1,2-dioleoyloxy-3-(trimethylammonium)propane; and PEOx represents polyethyloxazoline.*

**FIGURE 1:**
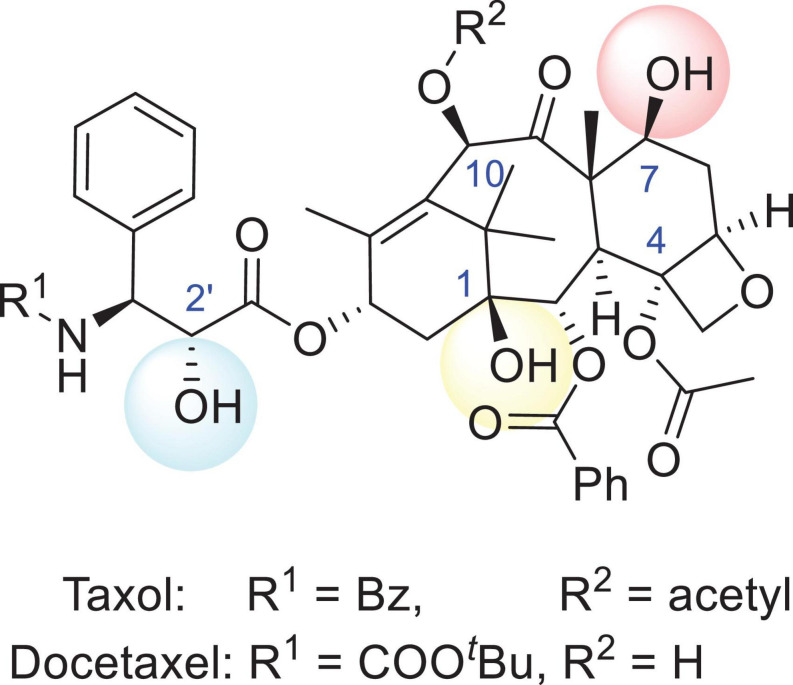
The structures of Taxol and docetaxel. The R^1^ group of Taxol (PTX) is *Bz* and the R^2^ group is *acetyl*, while the R^1^ group of docetaxel (DTX) is *COO^*t*^Bu* and the R^2^ group is a *hydrogen*.

Structure–activity relationship (SAR) studies of PTX have been systematically undertaken in the past three decades and are emphasized in the following sections (shown in Figure 1). Removal or replacement of either the hydroxyl group attached to carbon 1 (1-hydroxyl group) or the 2-acyl and 4-acyl groups of PTX reduces the bioactivity. The oxygen-containing heterocyclic ring is the pharmacophore. Notably, the 7-hydroxyl, 10-acetyl, and acetyloxy groups are the least important groups, so these regions can be further modified for multifunctional group introduction or removal. Reduction of the 9-carbonyl group leads to a slight enhancement in bioactivity. Hydroxyl or hydrolyzable ester groups should be bonded to carbon 2′, while phenylation or arylation requires linkage to carbon 3′. In addition, carbon 3′ must bear an amide group (Georg et al., 1992; Wahl et al., 1992; Georg and Cheruvallath, 1994; Kingston, 1994; Nicolaou et al., 1995; Gunatilaka et al., 1999). Although PTX has a bright future in chemotherapy, some inherent properties, including its poor aqueous solubility (≤0.4 M), have brought about many side effects *in vivo*. Aiming to identify analogs with better aqueous solubility, French pharmaceutical giant Sanofi developed the second-generation antitumor agent docetaxel (DTX) in 1996, which is structurally related to PTX. Compared with PTX, hydroxyl groups instead of acetyl or acetyloxy groups were tied to carbon 10 in DTX to increase the aqueous solubility. The benzoxy group on the amide side chain adjacent to the 3′ carbon was converted into tert-butyl carbamate (shown in Figure 1). Additionally, the formulation of PTX commonly used in clinical settings is diluted with polyoxyethylated castor oil (Cremophor EL) in a 1:1 v/v mixture with absolute ethanol before intravenous administration. Notably, a solubilizer combination composed of polysorbate 80 and alcohol used for DTX administration has been shown to be less susceptible to producing an allergic response, so doctors have usually tended to recommend using DTX once irritation occurs (Hanauske et al., 1992; New et al., 1996).

## Antitumor Efficacy of PTX

The first study on the anticancer activity of PTX was reported in 1971 (Wani et al., 1971), and a more in-depth study surrounding the anticancer mechanisms of PTX was disclosed by Schiff et al. (1979). Compared with another tubulin inhibitor, PTX inhibited the growth of cancer cells by promoting α- and β-tubulin subunit assembly in microtubules rather than microtubule dissociation, blocking cell cycle progression and mitosis. It was demonstrated that PTX promoted tubulin aggregation at lower critical concentrations and then activated the spindle checkpoint (mitotic checkpoint), resulting in cell division termination at either the G2 or M stage (Rowinsky et al., 1990; London and Biggins, 2014; Zierhut et al., 2019). Additionally, PTX participates in regulating cell functional responses, such as intracellular organelle transport and intracellular signaling pathways (Honore et al., 2005). For instance, the cytotoxic effects of PTX are predominantly achieved by apoptosis induction, which accelerates the production of early reactive oxygen species (ROS) and hydrogen peroxide (H_2_O_2_) in cancer cells (Ramanathan et al., 2005; Alexandre et al., 2006). Alexandre and his collaborators found that after the addition of a quantitative amount of PTX to MCF-7 cells (a human breast cancer cell line), the intracellular O_2_ concentration did not change within 8 h, while the extracellular O_2_ and H_2_O_2_ concentrations increased significantly. In addition, they found that PTX had a significant effect on the levels of ROS in HL-60 cells (an acute leukemia cell line) and mitochondrial defective HL-60/C6F cells (Alexandre et al., 2007). Other studies have also shown that PTX participates in regulating calcium signals and miRNA expression to induce cancer cell apoptosis and tumor therapy (Blagosklonny et al., 1997; Giannakakou et al., 2001).

Chemotherapy resistance (CR) is a major problem in cancer treatment, and, to a great extent, the cause of death from breast cancer cases can be ascribed to recurrence and metastasis (Chi et al., 2019). Long-term use of PTX might increase the odds of CR, leading to chemotherapy failure. More importantly, the underlying mechanisms of CR are still not well understood. Many possibilities have been speculated to be responsible for breast cancer resistance to PTX (Wang et al., 2014). However, overexpression of drug efflux proteins might be one of the key points. For example, excess expression of P-glycoprotein (P-gp) causes efflux pumps to extrude PTX from inside cells to the external environment, reducing PTX cytotoxicity toward cancerous cells (Wang Y. L. et al., 2018). Additionally, cancer cells can become resistant to PTX, which is possibly related to the high expression of spindle assembly checkpoint (SAC) and microtubule proteins (MAPs, such as MAP4), because the low expression of microtubules can cause the microtubules to become more fragile, making breast cancer cells more sensitive to PTX (Kavallaris, 2010). Another study showed that PTX resistance was associated with particular miRNAs. For example, a high expression of miR-200C-3P promoted breast cancer cell resistance to PTX (Khongkow et al., 2016). Therefore, the dosage of PTX recommended for individual patients was a major problem. It was reported that treatment with a low concentration (10–200 nM) of PTX inhibited the formation of the mitotic spindle, activating the mitotic checkpoint for apoptosis enhancement (Jordan et al., 1996; Sorger et al., 1997). Conversely, treatment with a higher concentration (>200 nM) of PTX damages a large number of microtubules, leading to kinase activation and apoptosis promotion (Liu and Stein, 1997; Wilson et al., 1999).

**FIGURE 2:**
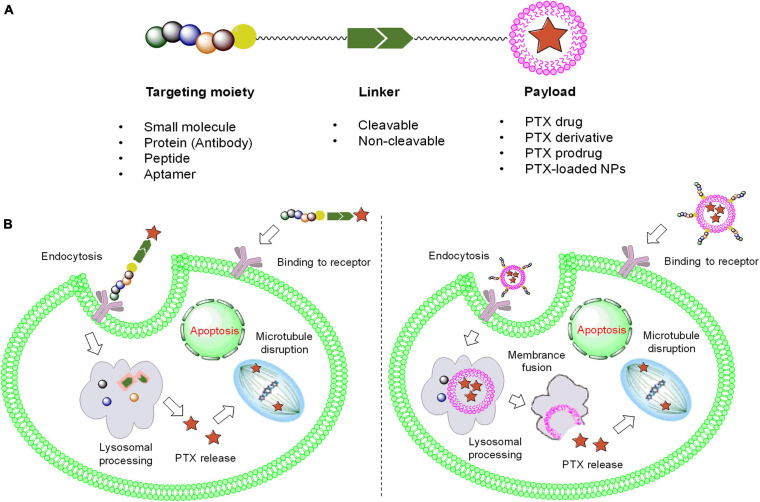
The framework and functional mechanism of ligand-PTX drugs. **(A)** The composition of ligand-PTX drugs. **(B)** Endocytosis of ligand–PTX conjugates and ligand-coated NPs with a PTX payload.

Although the use of PTX is prominent in clinical applications, some problems should not be ignored when seeking further chemotherapeutic options. PTX has extremely poor aqueous solubility, which results in low bioavailability, metabolic instability, high toxicity, and allergic reactions. Furthermore, injection of a PTX solution might result in neutropenia, bone marrow suppression, and even liver damage in many patients (Marupudi et al., 2007; Gornstein and Schwarz, 2014). Therefore, selecting the appropriate tumor-specific drug delivery method to package PTX as a prodrug or payload would reduce the side effects of PTX and improve the therapeutic outcomes to a great extent. As shown in Figure 2, common ligands, including small molecules, proteins (antibodies), aptamers, and other tumor-targeted strategies, have been introduced, and their tumor selectivity, penetration ability, circulation time, and respective limitations are described. Additionally, the linker (cleavable or non-cleavable) and payload [PTX or PTX-loaded nanoparticles (NPs)] both have a large impact on antitumor efficacy and specificity, which are mutually complementary to the therapeutic effect. In this review, comprehensive platforms for enhancing PTX specificity are discussed in detail, providing a promising outlook for PTX development in the future.

## Small Molecule-Mediated Targeting

**FIGURE 3:**
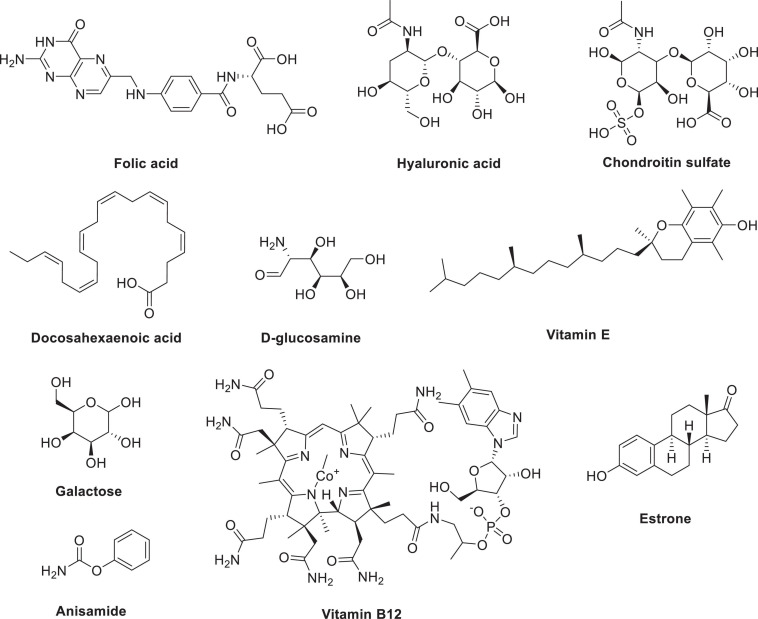
Commonly used small-molecule ligands.

The frequently used small-molecule ligands are shown in Figure 3. Due to the high affinity of ligands to their targets, small molecule-drug conjugates (SMDCs) have successfully enhanced the specificity of the cytotoxic payload toward tumors. SMDCs can distribute into tumor tissues rapidly with no immunogenicity (Srinivasarao and Low, 2017; Lee et al., 2018; Zhuang et al., 2019). In addition, SMDCs can quickly bind to the receptor and be cleared by the kidneys within 1 h (Srinivasarao and Low, 2017). The ligands are generally obtained by high-throughput screening, but this strategy involves a high cost and gives a low probability of success. In recent years, with the increase in DNA-encoded libraries, the availability of ligands has greatly improved. Although there have been no SMDCs approved by the FDA, some of these compounds have entered preclinical or clinical trials. For example, vintafolide [a vinblastine-folic acid (FA) conjugate], developed by Merck Sharp & Dohme Ltd. and Endocyte Ltd., failed in a phase III clinical trial, suggesting that SMDCs are still immature (Vergote and Leamon, 2015).

### Folic Acid (FA)

Folic acid receptors (FARs) are overexpressed in various cancers. In addition, FA plays a vital role in DNA synthesis, repair, and cell metabolism, thus making it an ideal ligand (Marchetti et al., 2014). FA-modified poly(lactic-co-glycolic acid) (PLGA)-based NPs were shown to exhibit 3.6-fold higher cell uptake than unmodified NPs *in vitro* (Luiz et al., 2019). Moreover, the PTX concentration originating from FA-functionalized PTX-loaded NPs (PTX-PEG-PLA-FA-NPs) was threefold higher than that of normal NPs and free PTX after SK-OV-3-bearing mice were treated with FA-NPs, NPs, and free PTX (Yao et al., 2018). Nano-formulations comprising PTX, PLGA, PEGylated octadecyl-quaternized lysine-chitosan (PEG-OQLCS), cholesterol, and FA showed remarkable anti-proliferative effects on HeLa cells (a human cervical cancer cell line) compared with Taxol *in vitro* and *in vivo* (Zhao et al., 2012). In addition, NPs coated with PEGylated folate sustained the enhanced permeability and retention effect (EPR), active targeting, and long circulation, while endogenous deoxycholic acid (DA) has been frequently used for adjusting the lipo-hydro partition coefficient of FA-NPs (Shen et al., 2012; Li L. et al., 2018). Combined with tumor microenvironment (TME)-responsive bonds (such as pH, redox, and enzymatic), FA-NPs were likely to increase drug accumulation to a large extent (Puvvada et al., 2015; Fan et al., 2020). Indeed, PGA is biodegradable by cathepsin B, which is highly expressed in tumor tissues (Decock et al., 2008).

The novel candidate PTX poliglumex (OPAXIO^TM^) is entering phase III clinical trials to evaluate its therapeutic efficacy against ovarian cancer (Galic et al., 2011). It was found that PTX-loaded liposomes incorporated with glutamic hexapeptide-FA (Glu_6_-FA) derivatives significantly affected microtubule stabilization as well as the cell cycle and cell migration (Yang et al., 2020). Aiming to improve the therapeutic efficacy, different drugs containing PTX, such as metformin (MET), doxorubicin (DOX), tariquidar (TQR), tanshinone IIA, and sorafenib, could be co-encapsulated by FA-functionalized vehicles to enhance the synergistic effects or reverse multidrug resistance (MDR) (Zhu et al., 2017; Xiao et al., 2018; Lei et al., 2019; Li et al., 2020; Zhong et al., 2020). Likewise, the combination of PTX and a photosensitizer, combining chemotherapy and photodynamic therapy, led to favorable targeting capability. PTX@FA-NLC-PEG-Ce6 nanocarriers composed of PTX, FA, and chlorin e6 (Ce6) showed great anticancer activity both *in vitro* and *in vivo* (Zhang Q. et al., 2019). In addition, various kinds of FA-coated NPs with PTX payloads, including micelles, microbubbles, nanofibers, gold NPs, gelatine-oleic NPs, nanovesicles, and graphene oxide, have been shown to enhance apoptosis induction and tumor growth inhibition (Tran et al., 2014; Wu et al., 2015; Mo et al., 2016; Luo T. et al., 2017; Liaskoni et al., 2018; Lv et al., 2018; Vinothini et al., 2019). To maximize the permeability, cell-penetrating peptides (dNP2, etc.) could also be incorporated into FA-NPs to facilitate deep penetration of the nano-formulations into glioma tumors (Li M. et al., 2018). However, a suitable FA ligand density has a significant effect on biological properties. FA-F127-PCL NPs with 10% FA showed superior cellular uptake and antitumor capability compared with NPs with 50 and 91% FA (Gong et al., 2019).

### Hyaluronic Acid (HA) and Chondroitin Sulfate (CS)

The CD44 receptor is often overexpressed on many malignant cancer cells (Platt and Szoka, 2008). Hyaluronic acid (HA), a widely used targeting ligand for the CD44 receptor, is inexpensive, easily conjugated, biocompatible, biodegradable, and non-immunogenic and has gained much attention worldwide (Luo et al., 2016). More importantly, HA-functionalized NPs exert a bystander effect, causing deep penetration and neighboring cell uptake in 3D tumor models (El-Dakdouki et al., 2013). Nano-complexes harboring composite NPs (mPPHP NPs), mPEG-PLA, and HA-conjugated PTX have exhibited preferable anticancer efficacy and tumor-targeting capabilities; moreover, a significant reduction in liver accumulation was observed compared with free PTX (Luo et al., 2020). Biodistribution studies have shown that compared with Taxol, FA-coated PLGA NPs (PTX-HA-PLGA NPs) had almost sixfold higher PTX concentrations in tumors (Wu et al., 2016). The HA-functionalized formulations (liposomes, etc.) were rapidly internalized by cancer cells. Accompanied by PTX preloading, the NPs induced strong tumor apoptosis, cellular arrest, and cytotoxic activity (Ravar et al., 2016; Su et al., 2018; Zhao et al., 2018). After activation by the TME (pH, redox), HA-NPs exhibited great lysosomal release and antitumor efficacy (Yin et al., 2015; Liu et al., 2016, 2020; Zhong et al., 2016; Han et al., 2019).

Anti-MDR drugs (such as apatinib) co-delivered together with PTX have shown a favorable efficacy enhancement (Zhang et al., 2020). It was found that HA-functionalized vehicles with a PTX payload notably suppressed tumor invasion, migration, and proliferation, including micelles (HA-CA), selenium NPs (HA-Se@PTX), and mesoporous hollow alumina NPs (PAC-HMHA) (Thomas et al., 2015; Gao et al., 2019; Zou et al., 2019; Tang Y. et al., 2020). Previous studies have shown that HA-coated nanostructured lipids delivering PTX remarkably prolonged blood retention and tumor accumulation compared with Taxol (Yang et al., 2013). Similar to HA, chondroitin sulfate (CS) has a high affinity for the CD44 receptor. Micelles comprising retinoic acid, CS, and PTX successfully inhibited the expression of multiple proteins associated with Golgi metastasis, angiogenesis, invasion, and tumor growth *in vivo* (Li et al., 2019; Zhang M. et al., 2019).

### Docosahexaenoic Acid (DHA)

**FIGURE 4:**
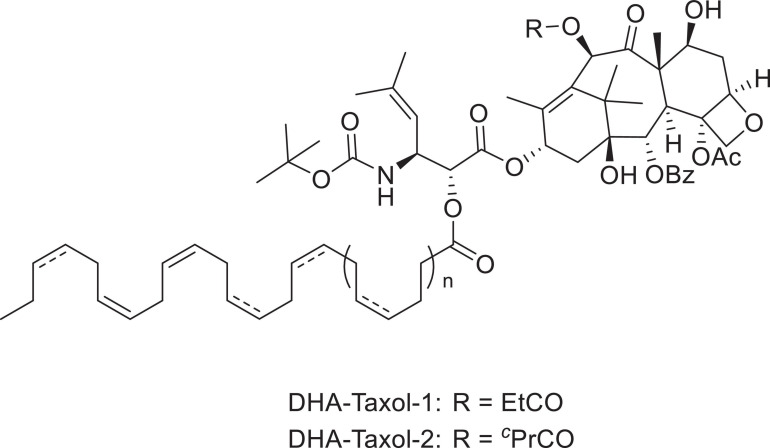
The structures of two DHA–PTX conjugates.

**FIGURE 5:**
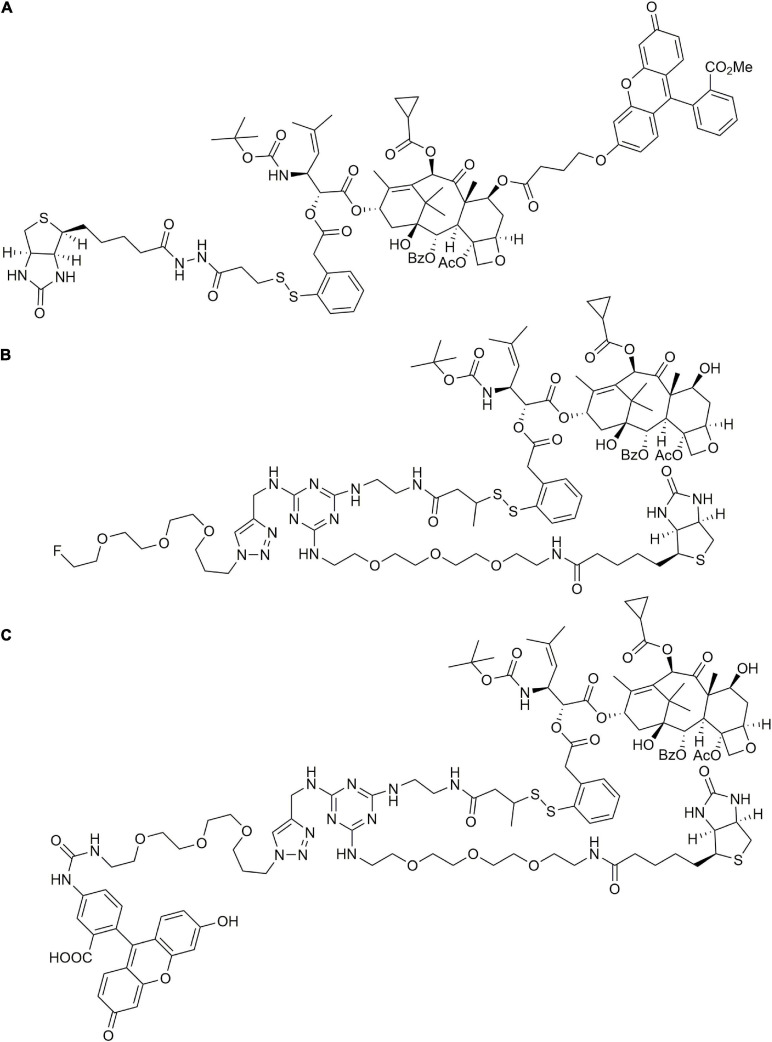
The structures of three biotin–PTX conjugates.

The Ojima group used tumor-targeting ligands such as docosahexaenoic acid (DHA), a polyunsaturated fatty acid, coupled with PTX to form DHA–PTX conjugates (shown in Figure 4). It was found that both conjugates DHA-Taxol-1 and DHA-Taxol-2 exhibited strong inhibition against DLD-1 xenografts and achieved total regression of drug-resistant and drug-sensitive tumors (Kuznetsova et al., 2006; Ojima, 2008). This group also designed fluorescently labeled redox-sensitive biotin–PTX conjugates, which were used as fluorescent probes to study the physiological and biochemical properties in biological samples and the mechanism of drug delivery through receptor-mediated endocytosis (RME) (Ojima et al., 2012) (shown in Figure 5A). Three years later, they introduced another two novel tumor-targeting conjugates bearing either a fluorine-labeled prosthetic as a potential 18F-positron emission tomography (PET) radiotracer or a fluorescent probe for internalization studies in a tumor-targeted drug delivery system *in vitro* (Vineberg et al., 2015) (shown in Figures 5B,C). Through assays, they found that in the presence of glutathione ethyl ester, only the fluorine-labeled prosthetic conjugate entered cells through the RME on the first day, exerting excellent cytotoxicity and selectivity.

### Glucoside-Based Molecules

Due to the exuberant metabolism of tumor cells, glucose transporters (GLUTs) are overexpressed on the surface of cancer cells, including lung, breast, colon, esophageal, head and neck, and thyroid cancers (Smith, 1999). Therefore, glucose is also able to act as a tumor-targeting ligand. It was found that D-glucosamine-functionalized copolymer NPs encapsulating PTX (DGlu-NP/PTX) exhibited more favorable anticancer ability and specificity than Taxol NPs and plain NPs without evident toxicity *in vitro* and *in vivo* (Jiang et al., 2014). In addition, the vast majority of the asialoglycoprotein receptor (ASGP-R) is expressed on the cellular membrane of hepatocytes and has often emerged as a specific target for hepatocellular carcinoma (Borker and Pokharkar, 2018). Owing to its multi-antennary galactose modification, these functionalized NPs were capable of binding to ASGP-R with an increased dissociation constant (K_d_). Thus, galactose-functionalized PTX-loaded gold NPs (Gal/PTX-GNPs) showed a considerable enhancement in anti-proliferative activity in HepG2 cells (a human hepatoma cell line) with little hepatotoxicity (Gao et al., 2017).

### Vitamin-Based Molecules

The overexpression of P-gp, an ATP-binding cassette transporter that pumps hydrophobic molecules out of the cell through the cellular membrane, is a key factor in MDR development (Amawi et al., 2019). D-α-Tocopheryl PEG-1000 succinate (TPGS), which is an amphipathic vitamin E (VE) derivative, has been considered an efficient P-gp inhibitor and is widely used for chemotherapeutic delivery (Collnot et al., 2010). NPs containing PTX and TPGS have been used for the treatment of various cancers, including lung, breast, colorectal, brain, and prostate cancer, among others (Gorain et al., 2018). For instance, it was found that 9-fluorenylmethyloxycarbonyl (Fmoc)-modified VE showed a strong interaction with PTX. Moreover, VE-based micelles (PEG_5__K_-VE_2_) encapsulating PTX exerted superior antitumor abilities with minimal adverse effects (Zhang et al., 2014). Due to the high expression of the CD320 receptor (CD320-R) in many cancers, vitamin B12 (VB12, a CD320-R ligand) is also frequently used in tumor-targeting therapy (Arora et al., 2017). Guo et al. (2019) demonstrated that VB12-sericin micelles with a PTX payload effectively penetrated tumor cells via CD320-R-mediated endocytosis to alter the mitochondrial transmembrane potential and cause tumor apoptosis activation.

### Other Small Molecules

Tenascin-C (TN-C), an extracellular matrix (ECM) glycoprotein, is highly expressed in various cancer cells, including ovarian, breast, and prostate cancer (Chiquet-Ehrismann and Chiquet, 2003). Sulfatide, an acidic sphingosaccharide that is partially sulfated, has a high affinity for TN-C. It was revealed that sulfatide-coated lipid perfluorooctylbromide NPs harboring PTX (PTX-SNPs) could prolong retention and accumulation, resulting in better tumor suppression than free PTX and non-specific NPs (Li et al., 2014). Likewise, alendronate (ALN) exhibits excellent bone-targeting abilities and is often adopted as an osteosarcoma ligand. ALN-grafted PTX-containing NPs displayed good cytotoxicity and tumor accumulation, resulting in preferable therapeutic efficacy over Taxol (Zhao et al., 2019).

In addition, the sigma-1 receptor, which is a membrane-binding protein that is highly expressed in human prostate tumors, is essential for prostate cancer cell survival and growth (Urandur et al., 2018). Anisamide has a high affinity for the sigma-1 receptor, which makes it a suitable ligand for prostate cancer treatment. It was revealed that amphiphilic micelles composed of PTX, anisamide, and *N*-octyl-*N,O*-maleoyl-*O*-phosphoryl chitosan (PTX-aM) could be triggered by low pH to increase cellular internalization and tumor accumulation and promote survival of PC-3 (human prostate carcinoma cell line)-bearing mice (Qu et al., 2020). Moreover, the estrogen receptor (ER) has a significant effect on the female endometrium, ovary, and breast, and upregulation of the ER has been observed in these tumor tissues (Borrow and Handa, 2017). Thus, estrone (ES), an estrogen, enables binding to the ER, wherein co-delivery of PTX and epirubicin was performed employing ES-functionalized liposomes. This nano-formulation exhibited increased systemic circulation and tumor growth suppression without evident toxicity (Tang H. et al., 2020).

## Protein-Mediated Targeting

### Albumin

**FIGURE 6:**
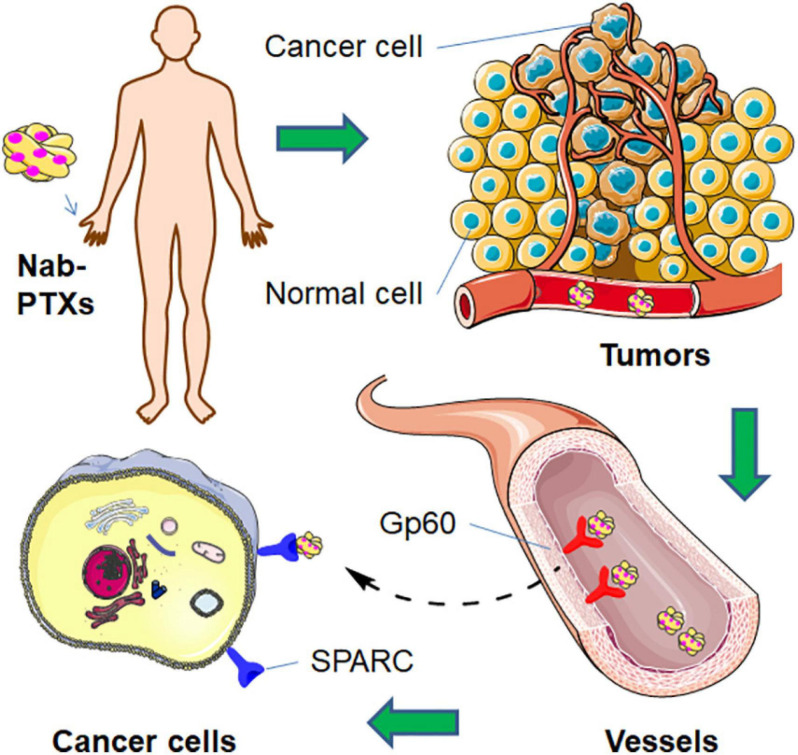
The endocytosis mechanism of nab-PTX.

Albumin is an endogenic serum protein. It accounts for over 60% of the total proteins in plasma and plays a very important role in various physiological functions (Jupin et al., 2013). Human serum albumin (HSA) has a preferential affinity for hydrophobic moieties and also shows biocompatibility, and thus, it has been exploited as a perfect delivery system for drug transportation. NP albumin-bound PTX (nab-PTX, Abraxane) is becoming the most important targeted chemotherapeutic drug for many types of cancer treatments. One kind of 60-kDa sialoglycoprotein (gp60) and secreted protein acidic and rich in cysteine (SPARC), an albumin receptor, are highly expressed on the surface of endothelial cells and cancer cells, respectively (Iglesias, 2009; Zhu et al., 2016). As shown in Figure 6, nab-PTX could first recognize gp60 in the endothelia and then activate the caveolin-1 process. After transportation into the tumor interstitium via the caveolae-mediated pathway, nab-PTX could bind to SPARC, facilitating the release of PTX around cancer tissues and improving the anticancer effects (Abu Samaan et al., 2019).

### Antibodies

Although some considerable progress has been devoted to SMDCs, their binding affinity is often questioned, causing non-negligible toxicity owing to off-target effects. Antibodies, known as immunoglobulins, are large Y-shaped proteins mainly secreted by plasmocytes that play roles in the immune system to identify and neutralize pathogens such as bacteria and viruses. Antibodies exist in the plasma of vertebrates and on the surface of the B cell membrane (Litman et al., 1993). One characteristic of antibodies is that they can recognize antigens via the fragment antigen-binding (Fab) variable region, which has derived many antibody-type anticancer drugs evaluated from this aspect. It has been more than 100 years since the discovery of antibodies in the 1880s, and they have become popular worldwide. With the advent of humanized antibodies and bispecific antibodies, functional monoclonal antibodies have also become increasingly successful (Husain and Ellerman, 2018; Baker et al., 2020). After the worldwide COVID-19 outbreak, antibodies have become potential drug candidates for its treatment. Antibodies are capable of inhibiting coronavirus activity and protecting cells from damage by binding to this virus (Marovich et al., 2020). However, the administration of a single antibody often fails in cancer therapy. Antibody-drug conjugates (ADCs) perfectly combine monoclonal antibodies with an effective cytotoxic payload, making full use of the specific binding ability to the target of the former and overcoming defects such as the low efficacy of the former and the large side effects of the latter. ADCs usually consist of three components: an antibody, a linker, and a cytotoxin. The antibody guides the ADC, which can deliver the payload to cancer cells in a targeted way. The linker regulates the pharmacokinetics and pharmacodynamics of the conjugate by altering the polarity of the molecule. With these components, ADCs can effectively penetrate tumors by specific binding to antigens and then endocytose into cancer cells.

**FIGURE 7:**
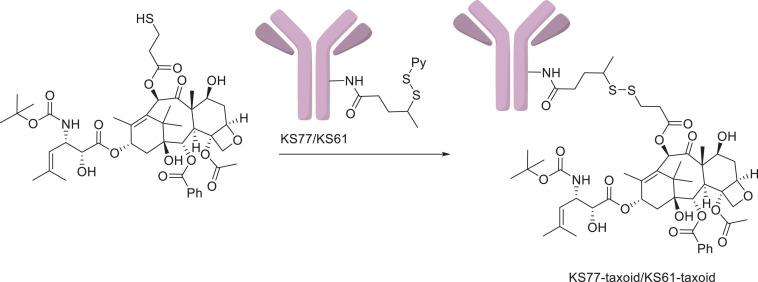
The KS77/KS61–PTX conjugates (Ojima et al., 2002).

**FIGURE 8:**
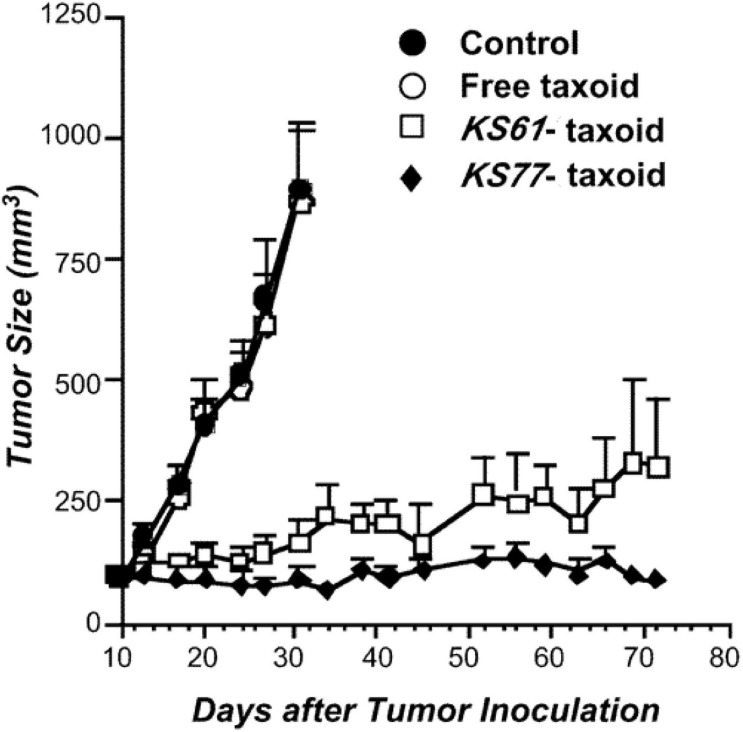
Antitumor activity of the KS77/KS61–PTX conjugates against A-431 xenografts in SCID mice (Ojima et al., 2002).

Paclitaxel, as a natural anticancer drug, bears great expectations for clinical applications, but its drawbacks, such as poor solubility and lack of tumor selectivity, are very prominent. Conversely, antibodies had low therapeutic efficacy but high aqueous solubility and excellent tumor target selectivity in cancer cells. Therefore, antibody–PTX conjugates appear to be a better choice for therapeutic applications that can garner the benefits of the two monomers. One of the earliest studies on antibody–PTX conjugates was by Guillemard and Saragovi (2001), who found that several PTX–antibody conjugates have high aqueous solubility, excellent tumor target selectivity, and high therapeutic efficacy, affording more cytotoxicity than free PTX or a mixture of free PTX and the free antibody *in vitro*. In addition, the PTX-ADCs prevented the growth of tumors and prolonged the survival of mice to a greater extent than free PTX. Ojima et al. (2002) used a disulfide linker to conjugate a PTX derivative and the antibody KS77 or KS61 (shown in Figure 7), which was specifically targeted to the epidermal growth factor receptor (EGFR). PTX-ADC was found to clearly prevent the growth of A-431 xenografts in SCID mice, whereas free PTX did not show any efficacy (similar to the blank sample) (shown in Figure 8). In 2010, the Safavy group designed a high drug-loaded completely soluble antibody–PTX conjugate linked with a discrete poly(ethylene glycol) (dPEG) for tumor-targeted chemotherapy. Through their experiments, they found that the conjugates remained homogeneous for a long time. The immunogenicity of these conjugates was also preserved via fluorescence-activated cell sorting (FACS) analysis compared with the sample without antibody. In addition, bioactivity studies in MDA-MB-468 cells (a human breast cancer cell line) proved that drug cytotoxicity was preserved (Quiles et al., 2010). This work provided a basis for further studies on antibody–PTX conjugates (Yao et al., 2016).

## Aptamer-Mediated Targeting

Aptamers, generated from the systematic evolution of ligands by exponential enrichment (SELEX) selection process, are three-dimensional structures of a single-stranded DNA or RNA oligonucleotide sequence with a short length of 20–100 bases and are considered to be monoclonal antibody mimics. Due to diversely combined sequences, these kinds of short oligonucleotides could form in unnumbered spatial conformations; thus, all aptamers interacting with target substances could be simulated and determined (Kong and Byun, 2013; Sun et al., 2016). SELEX has gradually become a hotspot in the research and development of biomaterials (Ni et al., 2020). Compared with monoclonal antibodies, aptamers have unique internal strengths, mainly as follows: high aqueous solubility, high stability, versatile modifications, high specific target binding affinity, ease of synthesis *in vitro* with no batch-to-batch variation, a low manufacturing cost, and low immunogenicity and toxicity. Since they are relatively quickly filtered by the kidney, general protocols for instillation of long-acting modifications have usually been carried out by chemical reactions to introduce macromolecules such as PEG to improve their renal threshold and half-life. Aptamers are used both alone for tumor treatment and as aptamer-drug conjugates (ApDCs) (Bouchard et al., 2010).

### AS1411

Nucleolin is located in the cell nucleus in normal cells, while immanent overexpression of nucleolin on the surface of various cancer cells has been observed (Abdelmohsen and Gorospe, 2012). AS1411 is a G-quadruplex-based aptamer that has been evaluated for its antitumor ability in phase II clinical trials (Rosenberg et al., 2014). This aptamer has shown favorable tumor specificity and biological compatibility and has been widely used in different drug delivery platforms. In addition, AS1411 itself has the potential to inhibit tumor-associated protein activity and may even be active in cell survival, growth, proliferation, nuclear transport, and transcription (Mongelard and Bouvet, 2010). Once AS1411 recognizes the targeted cell surface marker, the conjugates can penetrate the cells by endocytosis, and then the linker is cleaved, resulting in the release of the payload to the target site (Oh et al., 2014).

**FIGURE 9:**
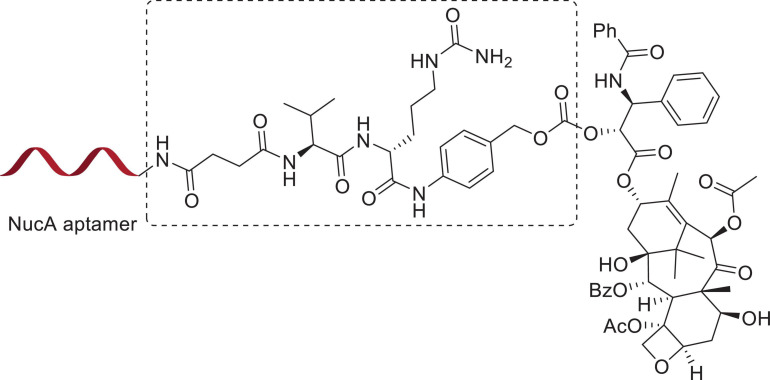
The structure of the NucA–PTX conjugate.

**FIGURE 10:**
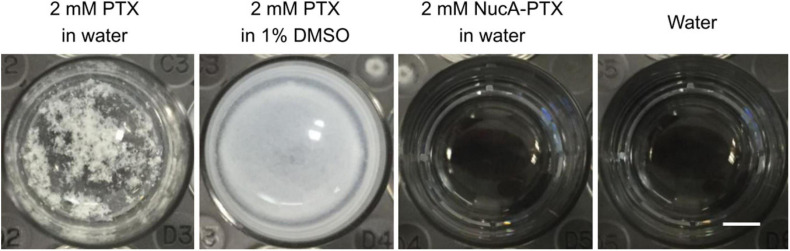
The aqueous solubility of free PTX and the NucA–PTX conjugate. The scale bar indicated is 3 mm. Copyright from Zhang et al. (Li F. et al., 2017).

In 2017, Zhang’s group conjugated the nucleolar aptamer and PTX with a stable cathepsin B-sensitive dipeptide linker to form an aptamer–PTX conjugate with high aqueous solubility, prodrug transmittability, and tumor inhibition (shown in Figures 9, 10), which gathered target-specific binding affinity derived from the aptamer and cytotoxicity generated by PTX. These ApDCs adopted a stable enzyme-cleavable dipeptide linker that prevented the side effects generated by PTX through off-target effects from normal cells. Only when cancer cells were recognized by the aptamer part of ApDCs could this drug enter the cell by endocytosis, and then the linker could be degraded by cathepsin B, resulting in the release of payload to the target site. Through experiments, they found that ApDCs afforded higher therapeutic efficacy and prevented the growth of mouse xenografted tumors compared with free PTX or the free aptamer *in vitro*, which demonstrated the anticancer ability of ApDCs to perform robustly and accurately (Li F. et al., 2017). Correspondingly, AS1411 incorporated into PTX-harboring NPs has frequently been used for antitumor therapeutic exploitation. For instance, Luo and collaborators described that AS1411-modified poly(L-γ-glutamyl-glutamine) nanoconjugates encapsulating PTX (AS1411-PGG-PTX) significantly increased cellular uptake, apoptosis, and anti-proliferative activity in U87MG cells (a human glioblastoma cell line). These NPs significantly increased tumor accumulation and extended the median survival time in glioma-bearing mice (Luo Z. et al., 2017). In addition, AS1411-coated PTX-loaded NPs (Apt-PTX-PLGA NPs) gathered benefits against glial cancer cells, with little impact on normal mammary epithelial cells. Aravind and his collaborators found high cellular uptake after GI-1 cells (a human glial cancer cell line) were treated with Apt-PTX-PLGA NPs in contrast to uncoated NPs. Visible retention, internalization, and tumor accumulation were observed *in vitro* and *in vivo* (Aravind et al., 2012). Additionally, it was demonstrated that AS1411-endowed HSA NPs carrying PTX (Apt-NPs-PTX) exerted preferential penetration via nucleolin-mediated endocytosis, resulting in increased anti-proliferative activity against MCF-7 cells, contrary to MCF-10A cells (a normal human breast cell line), which were barely affected (Wu et al., 2013). The combination of PLK1-targeted siRNA and AS1411-modified liposomes co-harboring PTX caused improved tumor cell apoptosis and anti-angiogenic efficacy *in vitro* and *in vivo* (Yu et al., 2019).

### Other Tumor-Targeted Aptamers

As mentioned above, PD-L1, MUC1, and heparin sulfate are upregulated in many cancers. In 2020, Zhang’s group developed a new PD-L1 aptamer–PTX conjugate that enhanced the anti-proliferative efficacy in PD-L1-overexpressing triple-negative breast cancer cells (Wu et al., 2020). In addition, Mie and colleagues designed novel targeting nanocomposites of elastin-like polypeptides, poly-aspartic acid, PTX, and MUC1-binding aptamers. These NPs displayed enhanced cytotoxicity to MCF-7 cells compared with non-targeted NPs (Mie et al., 2019). It was reported that a MUC1 aptamer conjugated to either PLGA NPs or chitosan-coated HSA with PTX encapsulation significantly increased the transmittability toward MCF-7 cells and T47D cells (a human mammary ductal carcinoma cell line) (Yu et al., 2011; Esfandyari-Manesh et al., 2016). HPA, which is highly expressed in human tumors, is responsible for heparin sulfate degradation. It has been proven that the S1.5 aptamer specifically binds to HPA (Silva et al., 2013). Therefore, S1.5-decorated PEG-PLGA micelles with a PTX payload [Apt(S1.5)-PTX-NP] exhibited favorable cellular uptake and cytotoxicity enhancement in MDA-MB-231 cells (a human breast cancer cell line) in comparison with plain micelles. In addition, Apt(S1.5)-PTX-NP significantly increased the anti-angiogenesis and anti-invasive effects, resulting in the most effective therapeutic efficacy (Duan et al., 2019).

Platelet-derived growth factor receptor β (PDGFRβ) is overexpressed in glioma and is involved in tumor angiogenesis, migration, and proliferation. It was found that the Gint4.T aptamer could tightly bind to PDGFRβ and transport across the blood–brain barrier (BBB), which is suitable for drug delivery to the brain (Delac et al., 2015; Monaco et al., 2017). Combined with glioma-targeting aptamer GMT8, dual-functionalized tetrahedral framework oligonucleotides carrying PTX were capable of inhibiting the migration, invasion, and proliferation toward U87MG cells, resulting in cancerous apoptosis enhancement (Shi et al., 2019). Additionally, Sun and collaborators demonstrated that RNA aptamers served as an effective tool against CD133^+^ cells. The targeted NP co-delivery of PTX and surviving siRNA (DP-CLPs-PTX-siRNA) resulted in selective apoptosis and tumorigenesis inhibition, leading to extended survival rates of glioma-bearing nude mice (Sun et al., 2018).

Non-small-cell lung cancer (NSCLC) represents a predominant class (>80%) of lung cancers, leading to significant cancer-related death worldwide. By virtue of SELEX technology, a novel aptamer (S15-APT) was developed, which exerted favorable affinity and specificity for NSCLC (Zhao et al., 2009). When S15-APT was incorporated into PTX-loaded NPs, an increased inhibitory rate was observed in various cancer cell lines compared with free PTX, including in HeLa cells, BEAS2B cells (a human bronchial epithelial cell line), and Caco-2 cells (a human colorectal adenocarcinoma cell line) (Engelberg et al., 2019). For prostate cancer with specific membrane antigen (PSMA) expression, A10-3.2 aptamer-functionalized PLGA-based nanobubbles delivering PTX (PTX-A10-3.2-PLGA NBs) showed high selectivity toward LNCaP cells (a human prostate cancer cell line). With the help of ultrasound, it was confirmed that these NBs achieved prominent tumor inhibition and increased median survival time without notable toxicity (Wu et al., 2017).

## Other Tumor-Targeting Strategies

### Magnetic Iron Oxide-Mediated Targeting

Magnetic NPs have been widely applied in the realm of cancer therapy owing to their physical properties and biocompatibility (Xie et al., 2010). It was reported that PTX loaded into poly(ethylene glycol) carboxyl-poly(ε-caprolactone) magnetic NPs (PEG-PCCL-MNP/PTX) induced excellent therapeutic ability with low phlebitis and hemolysis (Li X. et al., 2017). NPs composed of PTX and PEGylated PLGA-based superparamagnetic iron oxide NPs (PTX/SPIO-NPs) significantly enhanced the median survival time of glioblastoma-bearing mice (Ganipineni et al., 2018). Likewise, multifunctional magnetic NPs carrying PTX, SPIO, and Pluronic F127 suppressed tumor growth and prolonged the survival time of colon cancer-bearing mice (Dehvari et al., 2016). Combined with the pH-stimulating peptide H_7_K(R_2_)_2_, SPIO-based liposomes containing PTX [PTX/SPIO-SSL-H_7_K(R_2_)_2_] confirmed a good therapeutic effect on MDA-MB-231 cells *in vitro* and *in vivo* (Zheng et al., 2018).

### Biomembrane-Mediated Targeting

When PTX is coated with immune or cancer cell membranes, this drug has a fake identity to escape the clearance system and home to tumor regions. After pre-priming, engineered mesenchymal stem cells (MSCs) carrying PTX can release the drug package by secreting membrane microvesicles (MVs) into tumors for several days (Pascucci et al., 2014; Layek et al., 2018). In addition, cancer cells can produce extracellular vesicles (EVs), which are often used for delivery systems. It was demonstrated that lung cancer cell-derived EVs with the oncolytic virus and PTX payload importantly targeted the neoplasia and triggered inflammatory reactions (Garofalo et al., 2019). Additionally, E7-targeting siRNA and PTX co-loaded PLGA NPs camouflaged with HeLa cell membranes exhibited threefold higher accumulation than normal NPs in tumors. Clear synergistic anticancer abilities were observed, and the tumor volume ranges were reduced by almost 83.6% (Xu et al., 2020).

## Discussion and Future Perspectives

Since its discovery in the 1960s, PTX has become one of the most widely used chemotherapeutics throughout the world. Although 50 years has passed, the tremendous market of PTX, which has been a clinical and financial success, still attracts numerous pharmaceutical companies and scientists. Due to its poor solubility and severe adverse effects, such as hematotoxicity, neurovirulence, and gastrointestinal disorders, much effort has been made to overcome the drawbacks associated with its clinical application. Enhancing the tumor specificity of PTX is an effective solution to overcome these barriers. Currently, there have been many pathways that have achieved high affinity and specificity of PTX to tumors, mainly ligand-receptor-mediated targeting, cancer cell homing, and physics (superparamagnetism)-mediated guidance. Therefore, ligand-receptor-mediated active targeting has been studied in depth, and substantial progress has been made. According to the type of ligand (small molecules, antibodies, and aptamers) and payload (PTX and PTX-loaded NPs), tumor-specific PTX drugs can be simply classified into small-molecule–PTX (drug) conjugates (SMDCs), antibody–PTX conjugates (ADCs), aptamer–PTX conjugates (ApDCs), and ligand-functionalized PTX-loaded NPs.

In general, all ligand-PTX drugs consist of cytotoxic payloads, linkers, and tumor-targeting moieties. In recent decades, PTX SAR studies have been systematically undertaken and fully understood. The inactive groups could be either replaced for solubility improvement (DTX) or derivatized for targeting–moiety conjugation. At this point, ligand-PTX drugs have been enabled to sustain antitumor efficacy, and the cytotoxic payload does not have to be cleaved from the ligand, whereas high tumor specificity plays a vital role. On the other hand, modification of active sites would generate PTX prodrugs, which have minimized side effects on normal cells and tissues (Wang G. et al., 2018). However, the complete removal of modified groups has greatly contributed to PTX activation, emphasizing the importance of tumor sensitivity. Linkers can be mainly divided into cleavable and non-cleavable types. Particularly in ADCs, it is necessary to evaluate the residual effects when using non-cleavable linkers. TME-responsive linkers are frequently applied as connecting arms, accompanied by self-immolative spacers (Mccombs and Owen, 2015). When ligand-PTX drugs accumulate in tumors, the linkers are triggered by various stimulants in the TME and degraded. Afterward, self-immolative spacers can facilitate the release of PTX, resulting in an effective antitumor outcome. Thus, high tumor selectivity resulting from ligands is a critical point for therapeutic improvement.

Notwithstanding the capabilities of small molecules, antibodies, and aptamers toward tumor cells, these molecules have individual advantages and disadvantages. Small molecules have been adopted as tumor-specific ligands and exhibit great stability, rapid biodistribution, and high penetration abilities. However, their simple structures and low molecular weights (LMWs) often lead to inferior tumor specificity and kidney filtration compared with antibodies. Despite ADC succession on the market, inherent shortcomings, including instability, time-consuming preparation (>6 months), high immunogenicity, and manufacturing cost, significantly restrict the widespread use of antibodies. In contrast, aptamers composed of three-dimensional single-stranded oligonucleotides possess superior stability, easy synthesis and modification, high affinity and specificity, and low immunogenicity. More importantly, it is easy to screen specific ligands for a variety of targets utilizing SELEX technology. Recently, SELEX has become a hotspot for the recognition of undruggable targets. With the rapid development of screening processes, the new generation of SELEX relying on high-throughput sequencing, capillary electrophoresis, microfluidic chips, fluorescent magnetic beads, and so on has significantly reduced the selection periods (converted from 8–20 to 4 cycles), which provides bright prospects for individualized PTX-based treatment (Mendonsa and Bowser, 2005; Stoltenburg et al., 2005; Kim, 2011; Dupont et al., 2015).

According to current PTX applications in the clinic, it has been reasonably hypothesized that individual differences significantly affect the therapeutic efficacy and side effects among patients. Under transcriptomics and proteomics, it is easy to know whether CR-associated genes (such as P-gp) are highly expressed, affording a guideline for individual PTX-based therapy. Moreover, the burgeoning SELEX technology makes it possible to select personalized aptamers for each patient. It has been reported that primary cancerous cells and biopsies directly originating from patients have been used for individual tumor-specific aptamer selection (Liu et al., 2019; Takakura et al., 2019; Zamay et al., 2019). Additionally, organoid technology has become one of the most important breakthroughs, so patient-derived tumoroid-based SELEX is likely to exhibit exciting potential prospects for personalized ApDC treatment. Moreover, neoantigens caused by genetic mutations are perfect targets because of their existence only in tumors. Combining DNA/RNA sequencing, computational simulations, artificial intelligence, and SELEX technologies, complete tumor-specific aptamers are likely tobe obtained. A novel ApDC composed of a specialized aptamer and PTX is expected to eliminate off-target effects and significantly improve the therapeutic efficacy in patients with cancer.

## Author Contributions

YM and SY wrote the manuscript. SN, BZ, AK, and JG helped in revising the manuscript. AL and GZ proposed constructive discussions and supervised the manuscript. All authors contributed to the article and approved the submitted version.

## Conflict of Interest

BZ and AK were employed by the company Increasepharm (Hong Kong) Limited. JG was employed by the company Increasepharm (Hengqin) Institute Co., Limited. The remaining authors declare that the research was conducted in the absence of any commercial or financial relationships that could be construed as a potential conflict of interest.
